# Bipolar electrochemiluminescence at the water/organic interface†

**DOI:** 10.1039/d4sc06103a

**Published:** 2024-11-06

**Authors:** Yuheng Fu, Bingbing Xie, Miaoxia Liu, Shaojuan Hou, Qunyan Zhu, Alexander Kuhn, Lin Zhang, Wensheng Yang, Neso Sojic

**Affiliations:** a Engineering Research Center for Nanomaterials, Henan University Kaifeng 475004 China lin.zhang@henu.edu.cn wsyang@henu.edu.cn; b University Bordeaux, CNRS, Bordeaux INP, ISM UMR 5255 33607 Pessac France sojic@u-bordeaux.fr; c State Key Laboratory of Inorganic Synthesis and Preparative Chemistry, College of Chemistry, Jilin University Changchun 130012 China

## Abstract

Electrochemiluminescence (ECL) has emerged as a valuable tool for understanding multiphasic and compartmentalized systems, which have crucial wide-ranging applications across diverse fields. However, ECL reactions are limited to the vicinity of the electrode surface due to spatial constraints of electron transfer and the short lifetime of radical species, making ECL emission in bulk multiphasic solution challenging. To address this limitation, we propose a novel bipolar electrochemistry (BPE) approach for wireless dual-color ECL emission at the water/organic (w/o) interface. Firstly, amphiphilic glassy carbon (GC) microbeads with distinct hydrophilic and hydrophobic regions are prepared by bipolar electrografting of hydrophobic trifluoromethyl diazonium salt, then the resulting Janus beads are positioned at the w/o interface. Subsequently, two model ECL systems containing luminol and H_2_O_2_ in the aqueous phase, and [Ru(bpy)_3_]^2+^ and benzoyl peroxide (BPO) in the organic phase, are selected based on their solubility to confine light-emitting reactions to their respective phases. Upon application of an electric field perpendicular to the interface, the Janus microbeads get polarized, triggering simultaneous oxidative blue ECL (425 nm) and reductive red ECL (620 nm) in the aqueous and organic phases, respectively. Taking advantage of ECL imaging, the potential gradient distribution on the GC microbead at the w/o interface is revealed, indicating a “pseudo-closed” bipolar system due to limited ion transfer between phases. We also investigate the effect of changing the electric field direction parallel to the interface, which alters the ECL emission area from a hemisphere to a quarter of the microbead's surface. This bipolar ECL approach at the w/o interface not only offers opportunities for imaging the aqueous phase and organic phase simultaneously, but also enables ECL imaging and light generation in the bulk solution, thus overcoming the usual spatial limitation requiring proximity to the electrode surface.

## Abstract 2

A novel bipolar electrochemistry approach for wireless dual-color ECL emission at the water/organic interface is developed. This system overcomes spatial limitations of conventional methods, enabling imaging of complex multiphase environments.

## Graphical abstract

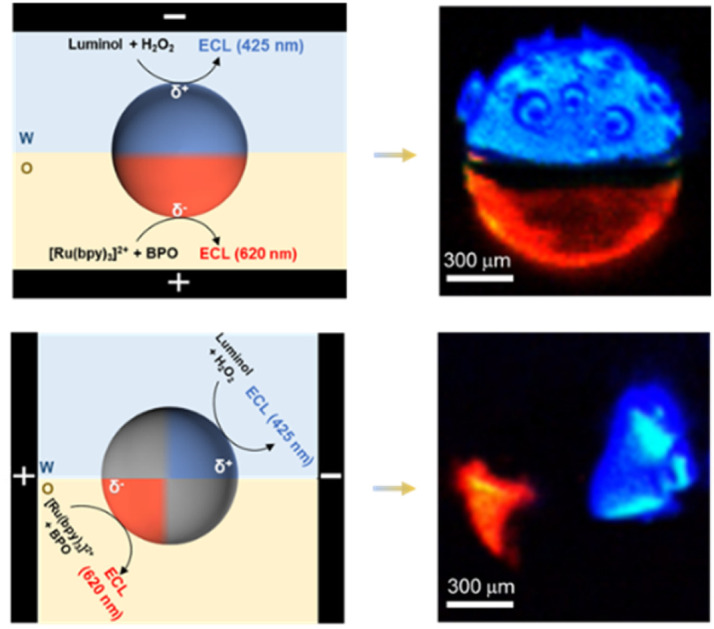

## Introduction

Electrochemiluminescence (ECL) is a process of light emission resulting from the electrochemical reaction of a luminophore, which forms excited species that emit light.^1,2^ Due to its high sensitivity, low background signal, and wide compatibility, ECL is well-established as a powerful analytical and microscopy technique with numerous applications in various fields, such as biomarker detection, cell imaging, chiral recognition, and dynamic monitoring.^3–5^ In addition to single liquid phase systems, ECL processes in multiphasic/compartmentalized systems are of great interest due to their potential applications in life science, for example, compartmentalization inside cells, not only by physical separation of molecular phospholipid layers, but also by liquid–liquid phase separation in nature.^6–8^ Therefore, establishing of ECL processes in multiphasic/compartmentalized systems is crucial for investigating complex and confined environments, as well as studying certain human diseases.^9^ In recent years, many efforts have been devoted to establishing ECL systems at water/oil (w/o) interfaces.^10–14^ Typical experimental setups involve w/o emulsion systems in contact with a working electrode surface. Bard *et al.* pioneered this field by reporting ECL of hydrophobic luminophores in oil-in-water. They utilized ionic liquids as both supporting electrolyte and emulsifier, achieving simultaneous oxidation of luminophore and co-reactant, leading to ECL emission within the emulsion upon application of a positive potential to the electrode.^15^ Later on, [Ru(bpy)_3_]^2+^ functionalized microgels are employed as emulsifier in an emulsion system, enabling the confinement of ECL emission at oil droplet interfaces and enhancing the localization of ECL reactions at the two-phase boundary.^16^ Subsequently, Dick's group developed a series of innovative studies. Taking advantage of the solubility differences between luminophore and co-reactant in different solvents, the ECL reaction is confined at the phase boundary, allowing for precise measurements of microdroplet contact radii, three-phase boundary thickness, and growth dynamics of electrogenerated O_2_ bubbles.^17^ Additionally, solvent entrapment within the aqueous microdroplet phase is visualized by ECL reaction using [Ru(bpy)_3_]^2+^ as the ECL luminophore and sodium oxalate as the co-reactant, leading to a clear contrast between the aqueous and the organic phase.^12^ When selecting a luminophore soluble in both phases and two co-reactants exclusively soluble in one phase or the other, phase-resolved mapping of liquid/electrode and liquid/liquid interfaces in multiphase systems is accomplished.^7^ Recently, the development of through-space ECL enables the observation of dynamic processes occurring far from the electrode surface, at distances up to hundreds of micrometers. By collecting reflected ECL light, this technique allows for the visualization of CO_2_ bubble growth, even when the bubble is located at a fraction of a millimeter from the electrode surface.^11^

Despite the successful application of ECL technology in exploring multiphasic systems, ECL emission at water–organic (w/o) interfaces remains largely confined to regions near the working electrode, due to the spatial constraints on electron transfer between the electrode and the solution, and the diffusion limitations arising from the short lifetime of radical species generated by co-reactants. Furthermore, conventional working electrodes employed in these multiphase ECL systems typically rely on either electrooxidation or electroreduction of a single type of luminophore and co-reactant. Consequently, ECL emission is often restricted to a single phase, making comprehensive imaging of two-phase systems challenging.

Bipolar electrochemistry has attracted significant attention due to its inherent “wireless” property and its ability to simultaneously trigger both electrochemical oxidation and reduction on a single electrode.^18–20^ This technique has been successfully applied to achieve wireless ECL for sensing and imaging applications.^21–24^ Recently, we report a bipolar electrochemical system at the w/o interface for the synthesis of Janus microbeads (Cu/GC, Au/GC, Cu/PANI, *etc.*).^25^ In this system, the w/o interface acts as a barrier, effectively separating the reactants. Under the influence of an electric field, amphiphilic GC microbeads located at the w/o interface become polarized, enabling simultaneous electrochemical oxidation and reduction reactions in the aqueous and organic phases, respectively. Building up on this concept, it is possible to establish wireless ECL systems based on microelectrodes stabilized at the w/o interface. Such systems could potentially facilitate two independent ECL processes occurring simultaneously in the aqueous and organic phases.

**Scheme 1:**
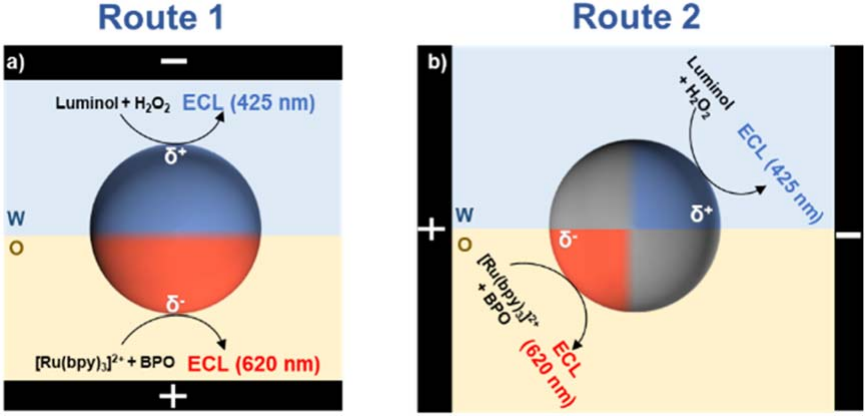
Illustration of the bipolar ECL emission by amphiphilic Janus particles at the w/o interface. Two orientations of the electric field are used: (a) perpendicular or (b) parallel to the w/o interface. The black rectangles symbolize the feeder electrodes. W and O indicate the water and organic phases, respectively.

In this work, we conduct bipolar ECL using amphiphilic Janus microbeads located at the w/o interface, as shown in Scheme 1. The polarized amphiphilic microbead triggers simultaneous electrochemical reactions in both phases upon applying an electric field, resulting in blue (425 nm) and red (620 nm) ECL emissions from the water and organic phases, respectively. This approach not only offers opportunities for imaging the aqueous phase and organic phase simultaneously, but also understanding multiphase systems by enabling ECL imaging and light generation in the bulk solution, circumventing the usual spatial limitations requiring proximity to the electrode surface.

## Results and discussion

To achieve bipolar ECL at the w/o interface, we first prepared amphiphilic GC microbeads capable of stabilizing at the interface between the two phases following our previously reported protocol.^25^ In brief, the amphiphilic GC microbeads with different ratios of hydrophilic to hydrophobic regions are prepared by bipolar electrografting of 3,5-bis(trifluoromethyl)phenyl diazonium salt on the polarized cathodic pole (*δ*^−^) of GC microbeads under the influence of appropriate electric fields. As illustrated in Scheme S1,† electrochemical oxidation of water occurs at the polarized anodic pole (*δ*^+^), while electrochemical reduction of the diazonium salt occurs at the polarized cathodic pole, leading to a partial surface functionalization of the GC microbeads with a hydrophobic layer. Importantly, it has been demonstrated that the electron transfer capability of the microbeads is not impeded by the grafting of this hydrophobic layer. Then the obtained amphiphilic Janus microbeads can be positioned at the w/o interface, with the hydrophilic part in the water phase and the hydrophobic part in the organic phase.

Two classical ECL systems emitting at different wavelengths in aqueous and organic phases are selected. The aqueous phase comprises luminol and H_2_O_2,_ while the organic phase contains [Ru(bpy)_3_]^2+^ and benzoyl peroxide (BPO). These model ECL systems are chosen due to their inherent solubility, luminol and H_2_O_2_ largely remain in water, whereas [Ru(bpy)_3_]^2+^ with PF_6_^−^ counter-ion and BPO primarily stay in dichloroethane. Consequently, the ECL reactions are confined to their respective phases. Scheme S2† outlines the mechanisms of the reactions involving luminol and H_2_O_2_ in the aqueous phase, and [Ru(bpy)_3_]^2+^ and BPO in the organic phase. In aqueous alkaline solutions, luminol is oxidized at the electrode surface to form a reactive intermediate. This intermediate then reacts with O_2_˙^−^ to produce the excited state 3-aminophthalate dianion, which relaxes to the ground state and emits blue light (425 nm).^26^ In the organic phase, the ECL reaction involving [Ru(bpy)_3_]^2+^ and BPO follows the “reductive-oxidation” pathway.^27–29^ In this process, [Ru(bpy)_3_]^2+^ and BPO are reduced at the electrode surface, and the co-reactant forms a strong oxidative radical that oxidizes the reduced luminophore to generate the excited state. This excited state returns to the ground state, emitting red light (620 nm).^7^

**Fig. 1:**
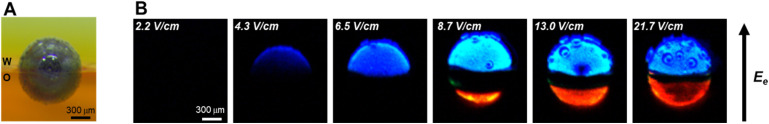
(A) White-light and (B) ECL images of amphiphilic Janus GC microbeads (*r* = 450 μm) at the w/o interface in the route 1 configuration. *E*_e_ represents the direction of electric field. (A) Image of the bead under white light before the application of the electric field. (B) Images in the dark of the ECL emitted by the bead when applying increasing electric field: 2.2 V cm^−1^, 4.3 V cm^−1^, 6.5 V cm^−1^, 8.7 V cm^−1^, 13.0 V cm^−1^, and 21.7 V cm^−1^. The amphiphilic Janus microbeads are pre-electrografted with hydrophobic 3,5-bis(trifluoromethyl)aniline, under the electric field of 9.0 V cm^−1^. The water phase contains 5 mM luminol, 50 mM H_2_O_2_ and 0.1 M NaOH. The organic phase consists of dichloroethane containing 1 mM Ru(bpy)_3_(PF_6_)_2_, 20 mM benzoyl peroxide and 20 mM tetrabutylammonium hexafluorophosphate. The distance between the two feeder electrodes is 2.3 cm.

Based on the established water-organic ECL systems, an amphiphilic GC microbead with equal hydrophilic and hydrophobic regions is chosen and stabilized at the w/o interface, as shown in Fig. 1A. Half of the bead is in the water phase and half in the organic phase. Then, an electric field perpendicular to the w/o interface (*i.e.* route 1 in Scheme 1) is applied to polarize the microbeads, ultimately triggering the electrochemical oxidation and reduction reactions in the aqueous and organic phases, respectively. Fig. 1B shows the original, unprocessed ECL images captured by the camera. As illustrated, with an increase in the electric field applied between the feeder electrodes, ECL from luminol (blue light) starts to appear in the region of the GC microbead located in the aqueous phase, while no ECL is observed in the region located in the organic phase. Indeed, ECL of luminol requires a lower anodic potential than the cathodic ECL system (*vide infra*). Under the electric field of 4.3 V cm^−1^, only this extremity of the bead emits blue light, but this ECL-emitting region extended further towards the middle of the bead as the polarized region increases.^30–32^ When the electric field increases to 8.7 V cm^−1^, red ECL from [Ru(bpy)_3_]^2+^ starts to appear at the cathodic extremity of the GC microbead situated in the organic phase, and simultaneously, the blue ECL in the aqueous phase becomes brighter. With the increase in electric field from 8.7 V cm^−1^ to 13 V cm^−1^ and 21.7 V cm^−1^, the region of red ECL in the organic phase continues extending towards the w/o interface, the region of blue ECL remains constant, but bubbles can be observed, indicating that water oxidation occurs in the aqueous phase. The images of the microbeads before and after the ECL reaction were captured to confirm that the ECL reaction does not cause any noticeable movement of the microbeads and the position of the microbeads at the w/o interface remains stable throughout the experiment (Fig. S1†).

To understand the evolution of ECL signals as a function of the electric field, cyclic voltammograms of reactants and their corresponding ECL-potential curves in both aqueous and organic phase are recorded. As shown in Fig. S2,† ECL from luminol starts emitting at +0.2 V, indicating that luminol and H_2_O_2_ are oxidized at similar potentials (Fig. S2A†). However, the electrochemical reduction potentials of [Ru(bpy)_3_]^2+^ and BPO exhibit significant differences (Fig. S2B and C†). The electrochemical reduction of BPO already occurs at 0 V, but red ECL can only be observed upon reaching −1.8 V, where the electrochemical reduction of [Ru(bpy)_3_]^2+^ takes place (Scheme S2†).^27,33,34^ Therefore, the observed blue ECL in the aqueous phase at low electric fields can be explained by the coupling of the oxidation of luminol and H_2_O_2_ in the aqueous phase with the reduction of BPO in the organic phase, at a potential at which the reduction of [Ru(bpy)_3_]^2+^ is not possible (*i.e.* red ECL from [Ru(bpy)_3_]^2+^ is not yet generated). Consequently, the gradual emergence of red ECL with increasing electric field can be related to the onset of the electrochemical reduction of [Ru(bpy)_3_]^2+^. It is interesting to notice that the bipolar electrochemical system at the w/o interface might be considered as a “pseudo-closed” bipolar system due to the high ion transfer resistance between the two phases. However, the gradual shift of the red ECL region with increasing electric field corresponds more to the characteristics of an open bipolar system, which exhibits a potential gradient along the surface of the GC microbeads. Additionally, at high electric fields, the presence of a black region between the blue and red ECL regions is observed, which may correspond to the “interfacial width” of the w/o interface. The high electric field induces ion migration across the phase boundary, entraining solvent molecules and forming nano- and microemulsions at the interface. These emulsions may create a zone where ECL reactions are inhibited, manifesting as a dark band at the w/o interface. This non-emitting region may represent the “interface width” of the w/o boundary.^8,35,36^

**Fig. 2:**
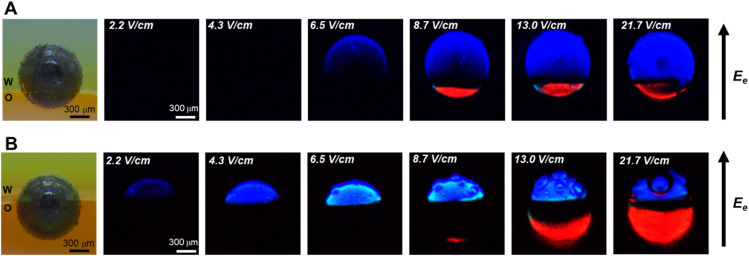
White-light and ECL images of amphiphilic Janus GC microbeads with different hydrophilic/hydrophobic regions at the w/o interface. The amphiphilic Janus microbeads are pre-electrografted with hydrophobic 3,5-bis(trifluoromethyl)aniline with different electric fields: (A) 4.2 V cm^−1^, (B) 19.2 V cm^−1^. Same experimental conditions as in Fig. 1.

To further study the bipolar ECL behavior of GC microbeads at w/o interface, two types of amphiphilic GC microbeads with unequal hydrophilic and hydrophobic regions are prepared. As shown in Fig. 2, the GC microbeads prepared at a lower electric field intensity during the grafting step have a smaller portion located in the organic phase, while those prepared at higher electric field have a larger portion in the organic phase when placed at the water/dichloroethane interface. Under the influence of an electric field oriented perpendicular to the w/o interface (*i.e.* route 1), both blue ECL in the aqueous phase and red ECL in the organic phase are triggered. The regions of blue and red ECL are determined by the initial hydrophilic and hydrophobic regions of the amphiphilic particles, respectively. This indicates a “closed” bipolar system character, with the point of zero polarisation located at the w/o interface, where mass transfer is hindered and the charges solely transfer through the bipolar electrode. Additionally, since the total net charge participating in bipolar oxidation and reduction at the two extremities has to be identical, the local current density varies with different active surface areas in the aqueous and organic phases, leading to higher ECL emission intensity with a smaller surface area and lower ECL emission intensity with a larger surface area. It can be observed that GC microbeads with a smaller portion located in the aqueous phase (Fig. 2B) exhibit higher blue ECL intensity compared to those with larger regions (Fig. 2A).

**Fig. 3:**
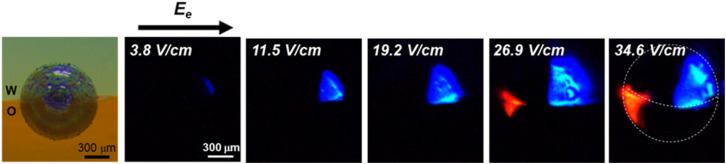
ECL images of amphiphilic Janus microbeads under electric fields of 3.8 V cm^−1^, 11.5 V cm^−1^, 19.2 V cm^−1^, 26.9 V cm^−1^, and 34.6 V cm^−1^. The direction of electric field indicated by the black arrow *E*_e_ is parallel to the water/dichloroethane interface (*i.e.*Scheme 1b). The amphiphilic Janus microbeads are pre-electrografted with hydrophobic 3,5-bis(trifluoromethyl)aniline under the electric field of 9.0 V cm^−1^. Half of the bead is in the water phase and the other half in the organic phase. Same composition of the water and organic phases as in Fig. 1. The dashed line indicates the boundary of the GC bead and the position of the w/o interface.

Taking advantage of ECL visualization, we have gained a clearer understanding of the potential gradient distribution on GC microbeads at w/o interface. The experimental results indicate that this system operates as a “pseudo-closed” bipolar system, due to limited mass transfer between aqueous and organic phase. However, it still exhibits potential gradients along the respective fractions of the particle, suggesting that mass transport is not entirely hindered across the liquid/liquid interface. Based on the experimental understanding of this BPE-ECL system, a possible physical schematic was proposed as shown in Scheme S4.† To further validate our hypothesis, we applied an electric field parallel, rather than perpendicular, to the w/o interface (route 2 in Scheme 1). The generated blue and red ECL serve as indicators reflecting the polarized potential gradient on GC microbeads at the w/o interface. As shown in Fig. 3, under a low electric field of 3.8 V cm^−1^, blue ECL begins to appear at the polarized anodic pole (*δ*^+^) immersed in the aqueous phase, with an enlarging emission zone as the electric field intensity increases. When the electric field reaches 26.9 V cm^−1^, red ECL starts to appear in the organic phase at the polarized cathodic pole (*δ*^−^) of the GC microbead, indicating that electrooxidation of luminol and H_2_O_2_ in the aqueous phase is coupled with electroreduction of [Ru(bpy)_3_]^2+^ and BPO in the organic phase. An interesting observation is that when the electric field is oriented parallel to the water/organic (w/o) interface, the previously observed black region diminishes, which is likely attributable to the substantial reduction or complete suppression of cross-interface ion migration. The ECL emission regions can be used to sense the influence of the electric field on the potential distribution and, consequently, the reactions occurring at the w/o interface. This approach provides valuable insights into the electrochemical processes taking place at the interface between two immiscible phases.

## Conclusions

We developed a new ECL system based on bipolar electrochemistry at the w/o interface. Amphiphilic Janus microbeads with tunable hydrophobic regions are synthesized using bipolar electrografting of a hydrophobic diazonium salt, followed by their strategic positioning at the w/o interface. Under electric fields oriented perpendicular to the w/o interface, these amphiphilic GC microbeads are polarized, triggering simultaneous electrochemical oxidation and reduction reactions in the aqueous and organic phases, respectively. By using specific single phase-soluble lumiphores and co-reactants, the ECL reactions are confined in their respective phases, resulting in blue ECL emission in the aqueous phase and red ECL in the organic phase. The regions of blue and red ECL are determined by the initial hydrophilic and hydrophobic regions of the amphiphilic particles, respectively, indicating a “pseudo-closed” bipolar system character. We also investigate the influence of the electric field orientation, demonstrating that aligning it parallel to the interface modified the ECL emission area from a hemisphere to a quarter of the microbead's surface. Compared to conventional methods, this approach offers unprecedented opportunities for simultaneous imaging of aqueous and organic phases, enabling ECL imaging and light generation in the bulk solution. It overcomes the usual spatial limitations requiring a positioning near the electrode surface, thus providing a powerful new tool for understanding complex multiphase environments. This work opens up new possibilities for applications ranging from multiphase catalysis to life sciences, increasing our understanding and control of interfacial phenomena.

## Experimental

### Chemicals

The following chemicals were used as received: 1,2-dichloroethane (Kermel, >99%), ammonium chloride (Kermel, ≥99.5%), sodium hydroxide ((NaOH), Macklin, ≥98%), luminol (Aladdin, 98%), benzoyl peroxide ((BPO), Macklin, 99%), tris(2,2′-bipyridine)ruthenium(ii) hexafluorophosphate (Ru(bpy)_3_(PF_6_)_2_, Bidepharm, 99%), 3,5-bis(trifluoromethyl)aniline (Aladdin, 98%), sodium nitrite (Sigma-Aldrich, ≥97%), hydrochloric acid (37%, Luoyang Haohua), dehydrated alcohol (Ante, ≥99.7%), tetrabutylammonium hexafluorophosphate ((TBAPF_6_), Macklin, 98%), hydrogen peroxide (Kermel, 30%), proton exchange membrane (Dupont, Nafion 115), anion exchange membrane (Fumasep, FAB-PK-130), and spherical glassy carbon (GC) powders (630–1000 μm) were purchased from Alfa Aesar.

### Apparatus

The surface treatment of GC electrodes was performed with a plasma cleaner (200 W, MiniFlecto). The electrochemical and ECL measurements were carried out with a photomultiplier tube (Hamamatsu, R5070A) and an electrochemical workstation (Autolab, PGSTAT101). White light and ECL images were captured using a digital camera (Tucsen, FL 20). Bipolar electrochemiluminescence experiments were carried out with a DC power supply (Elektro-Automatik, PSI 9200-25).

#### Three-electrode electrochemical system

The cyclic voltammetry measurements were carried out with a three-electrode system. A Pt wire was used as the counter electrode. The voltage of the PMT was set at 600 V and 550 V in the aqueous phase and organic phase solutions, respectively.

#### Bipolar electrochemical system

The bipolar electrochemical experiments were performed using three different cells. Cell 1 is for the preparation of the amphiphilic microbeads, while cell 2 and 3 are utilized for conducting the bipolar ECL experiments at the w/o interface.

##### Cell 1

The glass bipolar electrochemical cell is divided into three compartments by a proton exchange membrane and an anion exchange membrane. The positive feeder electrode is placed in the chamber separated by an anion exchange membrane, while the negative one is placed in the chamber separated by a proton exchange membrane. The distance between the two Pt mesh feeder electrodes (2 cm × 2 cm) is 2.5 cm.

##### Cell 2

Two square quartz tubes of different dimensions are combined to build the bipolar cell for ECL experiments at the w/o interface, as shown in Scheme S3A.† Two proton exchange membranes are used to separate the feeder electrode chambers from the bipolar reaction chamber. The feeder electrode chambers are filled with 250 mM PBS at pH 7. Two L-shaped Pt mesh feeder electrodes (1 cm × 2 cm) are positioned parallel to the w/o interface, with a distance of 2.3 cm between them.

##### Cell 3

The configuration of cell 3 is illustrated in Scheme S3B.† The quartz bipolar electrochemical cell consists of a central bipolar reaction chamber flanked by two feeder electrode chambers, with each compartment separated by proton exchange membranes. The feeder electrode chambers are filled with 250 mM PBS at pH 7. The distance between the two Pt mesh feeder electrodes (1 cm × 2 cm) is 2.6 cm.

#### Preparation of amphiphilic Janus microbeads

The reaction chamber of cell 1 was filled with a solution of 3,5-bis(trifluoromethyl)phenyl diazonium salt, containing 1 mM of 3,5-bis(trifluoromethyl)aniline, 10 mM hydrochloric acid, and 10 mM sodium nitrite. The two feeder electrode chambers were filled with an ethanol/water mixture (9 : 1, v/v), containing 0.2 M ammonium chloride. The GC microbeads (*d* = 900 μm) were placed in the reaction chamber of cell 1, after having been washed in deionized water under ultrasonication, and treated with plasma for 10 min. By applying different driving voltages of 12 V, 25 V and 55 V for 2 min between the feeder electrodes, the diazonium salt was electrografted onto the negatively polarized extremity of the GC microbeads.

#### Bipolar dual-color ECL at the w/o interface

The organic phase consisted of 1,2-dichloroethane containing 1 mM Ru(bpy)_3_(PF_6_)_2_, 20 mM BPO and 20 mM TBAPF_6_, while the aqueous phase contained 5 mM luminol, 50 mM H_2_O_2_ and 0.1 M NaOH. The amphiphilic Janus microbeads were positioned at the w/o interface. The bipolar ECL experiment with the electric field directed perpendicular to the w/o interface was conducted in cell 2, while the bipolar ECL experiment with the electric field directed parallel to the w/o interface was performed in cell 3, with the organic and aqueous phase solutions identical to those in cell 2.

## Data availability

The data supporting this article have been included as part of the ESI† and are available upon request to the authors.

## Author contributions

Y. Fu: investigation, methodology, analysis, writing – original draft; B. Xie: investigation, methodology, validation; M. Liu: investigation; S. Hou: investigation, methodology, validation; Q. Zhu: methodology, analysis; A. Kuhn: analysis, writing – review & editing; L. Zhang: conceptualization, supervision, funding acquisition, writing – review & editing. W. Yang: analysis, supervision, funding acquisition, writing – review & editing; N. Sojic: conceptualization, analysis, funding acquisition, writing – review & editing.

## Conflicts of interest

There are no conflicts to declare.

## Supplementary Material

SC-OLF-D4SC06103A-s001
