# Supplementary material for: Bipolar electrochemiluminescence at the water/organic interface
Source: Chem Sci. 2024 Nov 6;15(47):19907–12. doi: 10.1039/d4sc06103a (PMC11575568; doi:10.1039/d4sc06103a)
Supplement: SC-015-D4SC06103A-s001 [file SC-015-D4SC06103A-s001.pdf]

Supplementary Information (SI) for Chemical Science.
This journal is © The Royal Society of Chemistry 2024

Supporting Information

Bipolar Electrochemiluminescence at the Water/Organic Interface

Yuheng Fu,[a] Bingbing Xie,[a] Miaoxia Liu,[b] Shaojuan Hou,[a] Qunyan Zhu,[a] Alexander Kuhn,[b] Lin Zhang,[a] Wensheng Yang, *[ac] and Neso Sojic*[b]*

a. Engineering Research Center for Nanomaterials, Henan University, Kaifeng 475004, China. Email: lin.zhang@henu.edu.cn; wsyang@henu.edu.cn

b. University Bordeaux, CNRS, Bordeaux INP, ISM UMR 5255, 33607 Pessac, France. Email: sojic@u-bordeaux.fr

c. State Key Laboratory of Inorganic Synthesis and Preparative Chemistry, College of Chemistry, Jilin University, Changchun 130012, China.

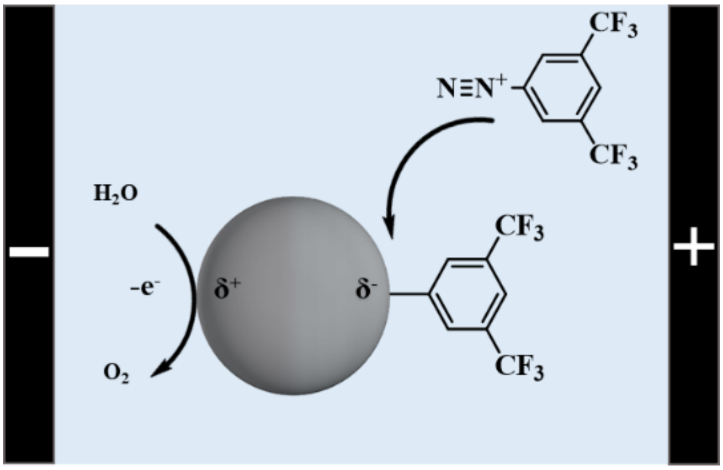

Scheme S1. *Preparation of amphiphilic Janus particles by bipolar electrochemistry.*

In **Scheme S1,** the amphiphilic Janus particles are prepared by bipolar electro-grafting a hydrophobic 3,5-bis(trifluoromethyl)phenyl layer.

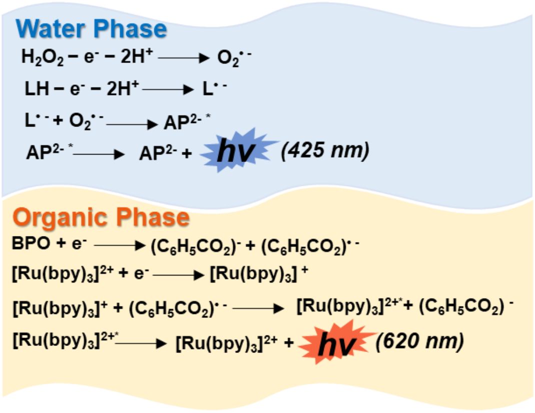

Scheme S2. *ECL mechanisms occurring at amphiphilic Janus particles located at the w/o interface operating in the water and organic phases, respectively.*[1–3]

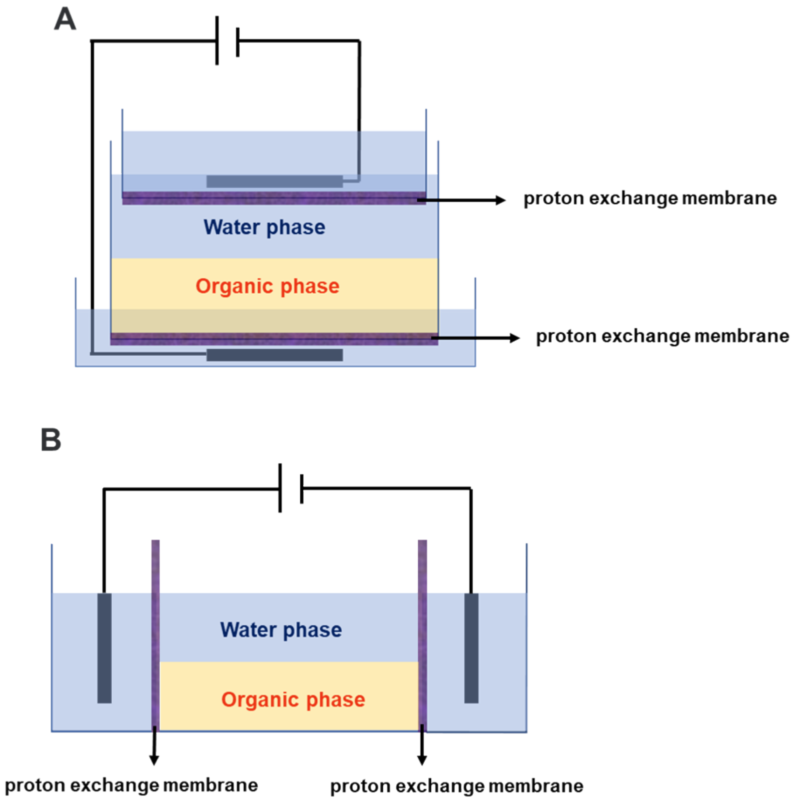

Scheme S3. *Schematic illustration of the bipolar ECL set-up with (A) Route 1: the direction of the electric field is perpendicular to the w/o interface (cell 2), (B) Route 2: the direction of the electric field is parallel to the w/o interface.*

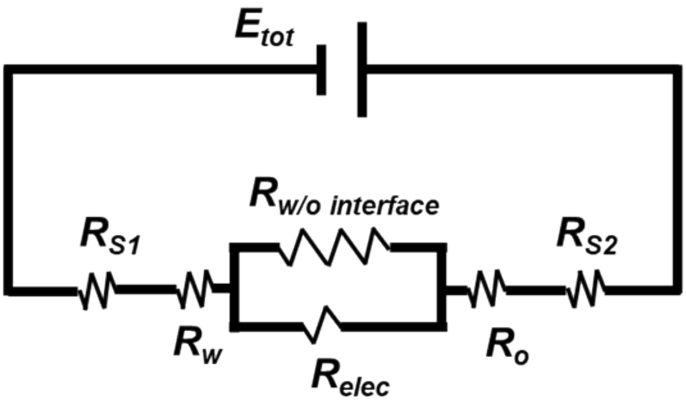

Scheme S4. *The physical schematics of amphiphilic microbead-base BPE-ECL system.*

Etot: applied potential, Rs1 and Rs2: resistance of the feeder electrode chamber solution, Relec: total resistance to electronic current posed by the bipolar electrode, Rw: resistance of water phase solution, Ro: resistance of organic phase solution, Rw/o interface: resistance of ions across the w/o interface.

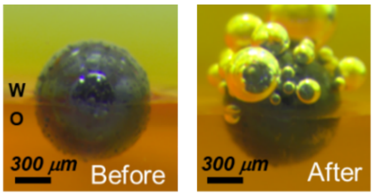

Figure S1. *Images of the Janus microbeads under white light before and after the bipolar ECL experiment.*

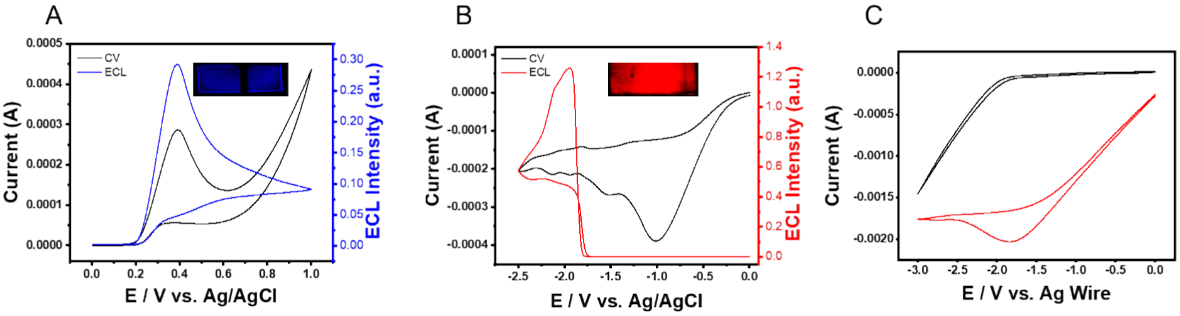

Figure S2. *Cyclic voltammograms and ECL intensity recorded with a GC plate (1*2 cm^2) (A) in aqueous solution containing 5 mM luminol, 50 mM H_2O_2 and 0.1 M NaOH. The scan rate was 100 mV/s. (B) in DCE containing 1 mM Ru(bpy)$_3$PF$_6$, 20 mM BPO and 200 mM TBAPF$_6$ under N_2 atmosphere. The scan rate was 100 mV/s. (C) Cyclic voltammograms recorded with a GC plate (1*2 cm^2) in DCE containing 100 mM TBAPF$_6$ with (red line) and without (black line) 20 mM BPO under N_2 atmosphere. The scan rate was 50 mV/s. Inset: optical images of ECL emission on GC plate under different conditions.*

References

1 S. Antonello, M. Hesari, F. Polo and F. Maran, *Nanoscale*, 2012, **4**, 5333–5342.
2 S. J. He, K. Chu, J. M. Wong, L. Q. Yang, J. R. Adsetts, R. Z. Zhang, Y. H. Chen and Z. F. Ding, *J. Anal. Test.*, 2020, **4**, 257–263.
3 A. B. Nepomnyashchii, R. J. Ono, D. M. Lyons, C. W. Bielawski, J. L. Sessler and A. J. Bard, *Chem. Sci.*, 2012, **3**, 2628–2638.
